# Preimplantation Genetic Diagnosis for Aneuploidy and Translocations Using Array Comparative Genomic Hybridization

**DOI:** 10.2174/138920212802510457

**Published:** 2012-09

**Authors:** Santiago Munné

**Affiliations:** Reprogenetics, 3 Regent Street, Suite 301, Livingston, NJ 07078, USA

**Keywords:** Array CGH, Translocations, PGD, PGS, Preimplantation genetic diagnosis, Down syndrome, Aneuploidy, Mosaicism, Chaotic mosaicism.

## Abstract

At least 50% of human embryos are abnormal, and that increases to 80% in women 40 years or older. These abnormalities result in low implantation rates in embryos transferred during in vitro fertilization procedures, from 30% in women <35 years to 6% in women 40 years or older. Thus selecting normal embryos for transfer should improve pregnancy results. The genetic analysis of embryos is called Preimplantation Genetic Diagnosis (PGD) and for chromosome analysis it was first performed using FISH with up to 12 probes analyzed simultaneously on single cells. However, suboptimal utilization of the technique and the complexity of fixing single cells produced conflicting results. PGD has been invigorated by the introduction of microarray testing which allows for the analysis of all 24 chromosome types in one test, without the need of cell fixation, and with staggering redundancy, making the test much more robust and reliable. Recent data published and presented at scientific meetings has been suggestive of increased implantation rates and pregnancy rates following microarray testing, improvements in outcome that have been predicted for quite some time. By using markers that cover most of the genome, not only aneuploidy can be detected in single cells but also translocations. Our validation results indicate that array CGH has a 6Mb resolution in single cells, and thus the majority of translocations can be analyzed since this is also the limit of karyotyping. Even for translocations with smaller exchanged fragments, provided that three out of the four fragments are above 6Mb, the translocation can be detected.

## INTRODUCTION

After fertilization *in vitro* human embryos are usually cultured for three days (cleavage stage) to 5 or 6 days (blastocyst stage). More than 50% of cleavage-stage embryos produced *in vitro* are chromosomally abnormal, increasing to up to 80% in women over 42 years of age [1-4]. Although some abnormal embryos arrest between day 3 and 5 [5], most do not, and even at the blastocyst stage more than 40% of embryos are abnormal increasing with advanced maternal age [6-8]. This is not limited to embryos resulting from super-ovulation cycles but also observed in natural stimulation cycles [9]. However, little is known of the frequency of chromosome abnormalities *in vivo*.

Most numerical chromosome abnormalities detected in cleavage and blastocyst embryos are not compatible with implantation or birth [10], which negatively affects the success of assisted reproductive treatments. The detrimental effect of aneuploidy is illustrated by the high prevalence of chromosome abnormalities detected in spontaneous abortions [11, 12], exceeding 70% in some studies of samples not cultured and analyzed by CGH or arrays [13-16]. 

Therefore the working hypothesis for Preimplantation Genetic Diagnosis (PGD) is that by selecting chromosomally normal embryos that have higher changes to survive to term, the success rate of assisted reproduction techniques can improve [17]. The use of PGD for this purpose is also known as Preimplantation Genetic Screening (PGS).

### Shortcomings of Pre-Array Technologies

PGS strategies first consisted of using fluorescence in situ hybridization (FISH) analysis with 5-10 probes of one or two cells biopsied from day-3 embryos (containing 4-10 cells) [17-21], trophectoderm cells biopsied from blastocyst stage embryos [22] or polar bodies biopsied from oocytes or zygotes [23-25]. These FISH methods were unable to provide a full chromosome complement count but the 12 probe assay was able to detect more than 80% of chromosomally abnormal embryos detected by array technology [26].

Initial studies utilizing the above approach reported an improvement in implantation rates, fewer spontaneous abortions and/or an increase in ongoing pregnancies [20, 27-38] but were not clinically randomized. In contrast, clinical randomized studies performed outside the initial centers that had developed the above protocols did not detect significant improvements or even showed a detrimental effect of PGS [39-42]. Several reasons for these conflicting results have been proposed but the most obvious one is technical differences between the two groups of studies.

Biological factors that could explain such differences are the occurrence of mosaicism and the potential self-correction of aneuploid embryos. Regarding mosaicism, many studies [1, 3, 4, 17, 43-48] have shown high rates of mosaicism in cleavage-stage embryos and thus any analysis based upon a single cell could be unreliable. That was more so in earlier studies using a few chromosome probes, but later studies using at least 8 probes showed that the majority of mosaic embryos display chromosome abnormalities in every cell. In such cases, the biopsied cell may not be chromosomally identical to the remaining cells of the embryo, but the clinical diagnosis of ‘abnormal’ will be valid. Large follow up studies of preimplantation embryos diagnosed using FISH estimate only a 5-7% error caused by mosaicism [20, 48] when embryos are reanalyzed in all their cells by FISH with 8 or more probes. When embryos are analyzed by array CGH the error rate measured by analyzing the remained cells of the embryo is merely 2%, and thus mosaicism is unlikely to be the primary cause of poor outcomes following PGS [49]. Instead, further analysis of aCGH embryos show that almost 100% of them have all their cells abnormal, but that could not be detected with a few probes in the initial FISH studies.

A second biological factor could be the self-correction of embryos. This hypothesis is based on several misconceptions. First is that placental confined mosaicism is caused by aneuploid embryos self-correcting into normal fetus and abnormal placenta. However, some studies now suggest that is the placenta that becomes abnormal [50]. Second, the culture of inner cell masses from embryos classified as abnormal by PGS to produce stem cells and consisting in culturing these cells through many passages, eventually results in the enrichment of normal cells [51, 52]. However, human embryos are replaced whole to the uterus and do not grow in monolayer, plus they do not go through so many passages before differentiating into tissues. In addition, according to Verlinsky *et al.* [53] abnormal embryos that self correct in monolayer had often started as normal zygotes that became abnormal postmeiotically (normal polar bodies, abnormal day 3 biopsy), and thus were already mosaics, than those that started as abnormal zygotes (abnormal polar bodies, abnormal day 3 biopsy).

An example of how little self-correction there is, is to compare the frequency of trisomy 15 at cleavage-stage embryos (1.87%) [10] with that of uniparental disomy (UDP) 15. If a trisomy-15 self-corrects by losing at random one chromosome 15, one-third of resulting embryos will have UPD. However, UPD-15 occurs in only 0.001% of newborns (OMIM, www.ncbi.nlm.nih.gov/omim), a mere 0.56% correction. 

One of the most probable causes of inter-center differences in PGD results is variation in the biopsy and genetic test used [58]. For example, one of the studies showing no improvement in IVF outcome following PGD biopsied two cells from each cleavage-stage embryo [39]. However, the same group later reported that two-cell biopsy, but not single-cell biopsy, is detrimental to embryo development [59]. 

Another study conducted by Mastenbroek *et al.* [41] reported an astonishingly high rate of diagnostic failure (20%), resulting in many embryos being transferred without a diagnosis. The implantation rate of these undiagnosed embryos was 59% lower than the control. In this case, the only difference between the control and test groups was that the test group was biopsied on day 3 of development, suggesting that embryo viability was drastically reduced by the biopsy procedures used in the clinics involved.

Preliminary evidence is now indicating that day 3 biopsy, even if well performed, could diminish implantation rates compared to controls [59, 60]. That may or may not be compensated in excess by PGD selection methods depending if they are performed correctly or not. Blastocyst biopsy seems now to be much less detrimental than day 3 biopsy [6, 61]. 

In addition of biopsy methods and skills, the “error rate” of the diagnosis technique is the second most important factor. The steps after biopsy involved fixation, FISH with a variety of different protocols and number of probes, and cell analysis. The overall accuracy of these steps is summarized in a single number, which is the error rate of a PGD laboratory. This error rate is obtained by analyzing all the cells of non-replaced embryos and determining if the original diagnosis was confirmed. Unfortunately error rates among different PGD laboratories range from 2-7% [20, 47, 49] to 40-50% [62, 63], with error rates around 50% in fact decrease implantation rates [58].

When performed using optimal methods, FISH can detect 91% of the chromosome abnormalities detectable by array CGH [21, 26], and in some laboratories did improve ART outcome. Regardless, the field of PGD is transitioning towards either polar body biopsy from oocytes or zygotes [64-66] or to trophectoderm biopsy from blastocyst stage embryos [6, 61]. 

Additionally, FISH is rapidly being replaced by comprehensive methods of DNA analysis, which detect close to 100% of numerical chromosomal abnormalities. These techniques are extremely redundant (each chromosome tested multiple times at different loci), automated, less subjective, and in some preliminary studies less prone to errors (2%)[49]. 

### Comprehensive DNA Analysis Techniques: Technical Differences

Comparative Genome hybridization (CGH) was first applied to day-3 embryo biopsies [67-71]. However, CGH is time consuming requiring the cryopreservation of embryos while testing is ongoing. When it was first applied embryo freezing had a low survival rate and embryo freezing and thawing neutralized any beneficial effects of PGD. CGH was abandoned for these reasons and not applied again until the development of vitrification [72].

In conjunction with vitrification, CGH has been clinically applied to polar bodies [64, 65, 73, 74] and blastocyst biopsies [6, 74]. 

Three other techniques, microarray CGH (aCGH) [75-78, 49], single nucleotide polymorphism (SNP) microarrays [79-81] and qPCR [89] are being used for PGD of single cells from PBs or day-3 biopsies yielding results in 24 hours. The rapid turnaround time for these methods eliminates the need to freeze embryos if the biopsy is done at those stages, and if the biopsy is done on day-5 and analyzed by aCGH or qPCR, the embryos can still be replaced on day-6 if the sample does not need to be flown in.

CGH, aCGH and SNP-microarrays rely on whole genome amplification (WGA) to amplify DNA from one or more cells from an embryo. According to our experience current WGA methods such as multiple displacement amplification (MDA), GenomePlex and PicoPlex allow for better overall coverage of the genome compared with earlier WGA methods (e.g. degenerate oligonucleotide primed PCR) that were more inclined to preferentially amplify some parts of the genome while leaving others unamplified or under amplified. 

The aCGH chip most commonly used (24sure, Bluegnome, Cambridge, UK) for the purpose of PGD utilizes about 4000 bacterial artificial chromosome (BAC) probes, 150,000 bp in length, covering all chromosome bands, covering ~25% of the genome sequence, and giving a resolution of about 4 MB or better than karyotyping. aCGH has a similar or higher accuracy rate as metaphase CGH, and should producing similar high implantation rates to those obtained with the older technique [6]. 

aCGH provides a quantitative analysis based on comparing the relative amount of test DNA (e.g. a blastomere) to the amount of known control DNA from a chromosomally normal individual. DNA from these two sources are differentially labeled and hybridized to probes on the microarray. The diagnosis is very accurate because multiple copies of each probe are placed on the microarray and each chromosome is tested at many distinct loci. 

Chromosome imbalances such as aneuploidies, unbalanced translocations, deletions and duplications are easily detected, but a limitation of aCGH is that diploidy cannot be distinguished from changes involving loss or gain of an entire set of chromosomes such haploidy or polyploidy. This is however a small limitation since 7.7% (n= 91,073) of 2PN embryos tested were polyploid or haploid but the majority of them had additional abnormalities detectable by aCGH and only 1.8% of all embryos were homogeneously polyploid or haploid. Furthermore, of those, the majority arrested by day 4, leaving only 0.2% of developing embryos uniformly polyploid or haploid that could produce a misdiagnosis [49, 82]. 

Single nucleotide polymorphisms are areas of the genome where a single nucleotide in the DNA sequence varies within the population. Most SNPs are biallelic, existing in one of two forms, and are found scattered throughout the genome. By determining the genotype of multiple SNPs along the length of each chromosome a haplotype (a contiguous series of polymorphisms on the same chromosome) can be assembled. This ultimately allows the inheritance of individual chromosomes or pieces of chromosomes to be tracked from parents to embryos. Current SNP microarrays simultaneously assay hundreds of thousands of SNPs and use software to distinguish how many copies of each chromosome there are in the sample [79-81].

The small size of the SNP array probes, can lead to poor hybridization efficiencies and low signal intensities for individual SNPs. This factor, coupled with the failure of WGA methods to amplify the entirety of the genome, can lead to many SNPs yielding no result. Also, allele drop out (ADO) and/or preferential amplification (PA) of one SNP allele versus another can lead to a great deal of ‘noise’ in the system. Three methods for cleaning up SNP-microarrays data have been developed: Qualitative methods, looking only at the inheritance of specific SNPs and requiring comparison with parental DNA samples; quantitative approaches, assessing only the intensity of SNP calls; and a combination of quantitative and qualitative methods. 

Qualitative approaches require the assessment of parental DNA to deduct the four parental haplotypes for each chromosome. Embryo testing is then focused on detecting the individual parental haplotypes, revealing how many chromosomes were inherited from each parent [79]. This approach has the disadvantage that mitotic abnormalities, in which only two haplotypes are present in a trisomy will not be detected. 30% of human embryos contain mitotic abnormalities or mosaicism [20]. The argument that this is actually an advantage because mosaics can lead to misdiagnosis is incorrect since through aCGH only 1.8% of embryos are misdiagnosed due to mosaicism [49]. This is because most mosaics, once all chromosomes are analyzed contain only abnormal cells and thus any single cell will allow a diagnosis of abnormal to be made. 

A quantitative approach compares the intensity of each SNP against the other SNPs and for aneuploidy screening it may not require parental testing. This approach is currently the least developed. 

A qualitative/quantitative approach has also been applied clinically, and probably can obviate the issues mentioned above for purely qualitative or quantitative approaches [80, 81]. All SNP analysis approaches have the limitation that it cannot detect postmeiotic tetraploidies since only two haplotypes are present. 

SNP-based microarrays offer some minor advantages over aCGH. One is that qualitative analysis SNP-based microarrays permits the detection of the parental origin of chromosome abnormalities. This is of little relevance to cases of advanced maternal age where at least 90% of the aneuploidies will be maternal in origin. Paternal origin aneuploidies are most likely mitotic error where the abnormal chromosome was randomly recruited as the extra chromosome. These errors offer no predictive value for other embryos in the cohort or for future cycles. 

For PGD of translocations SNP microarrays can differentiate between normal and balanced (carrier) embryos. However, because the rate of abnormalities in translocation cases is generally very high (>80%) [83], the majority of PGD cycles do not have a surplus of embryos that will allow choosing between normal and balanced embryos to be replaced. 

Although SNP arrays can produce a fingerprint of the embryo to determine which embryo implanted if more than two were replaced and only one implanted. However, fingerprinting can also be performed after aCGH by utilizing a small aliquot of the DNA produced by WGA to perform conventional DNA fingerprinting.

Finally, another potential advantage of qualitative SNP arrays is that it can also detect uniparental disomy (UDP), but this is a very rare event (e.g. uniparental disomy 15 occurs in 0.001% of newborns (OMIM)) and most embryos with UDP are chaotic mosaics with other abnormalities. 

A disadvantage of a qualitative or combination approach to SNP array analysis is the need to assess parental DNA ahead of the PGD cycle. This complicates patient management, adds to the cost of the test, and complicates ad hoc decisions regarding to perform PGD or not. Since approximately 20% of IVF cycles with planned PGD are cancelled on day three due to low embryo numbers, these couples would have spent money on pre-cycle parental testing that was at the end unnecessary. A summary of the differences between techniques can be found in (Table **1**).

### Validation of Microarray Methods

Due to the intrinsic and often unforeseen problems with a new technology, a new method should be validated against a more established one [84]. Assessing a new approach against itself may preclude the detection of technique related flaws. Thus, some inadequate validation methods are the analysis of cell lines with defined chromosome abnormalities which cannot mimic chaotic mosaicism [81]; analysis of eggs or embryos by one technique with analysis of polar bodies or the remainder of the embryo by the same technique which will preclude identifying abnormalities not detectable by that technique [66]; replacing undiagnosed embryos blindly and following pregnancies and clinical losses to determine the fate of each tested embryo does not account for the status of non-implanted embryos; or using the SNP calls in one chromosome as internal controls for other SNPs in that same chromosome [81]. In addition, the use of analysis tools that are qualitative in nature will miss the presence of two chromosomes of the same grandparental origin caused by mosaicism.

An optimal method for validating a new technique is to reanalyze those embryos that were not transferred to the patient, either because they underwent arrest or because they were diagnosed chromosomally abnormal, and the reanalysis of such embryos should be done with another well established technique to discern shortcomings of the new method under evaluation. The only problem with this approach is the scarcity of non-replaced normal embryos.

SNP-microarrays have undergone a variety of validation experiments [80, 81, 84, 85] but no studies so far have confirmed the original diagnosis by reanalyzing the remaining embryonic cells with a different technique. 

Microarray-CGH for PGD has been validated by analysis of single cells from known cell lines (Dagan Wells, personal communication) and by analyzing oocytes and their respective polar bodies both by aCGH [66]. The best validation study consisted of the single blastomere analysis by aCGH followed by reanalysis in all or most of their remaining cells by FISH using 12 probes plus probes for any chromosomes found abnormal according to aCGH. Only 1.8% of embryos were found to be incorrectly diagnosed [49]. 

qPCR has just been validated using a non-selection study [96], in which embryos were biopsied either on day 3 or at blastocyst stage, the biopsy analyzed by qPCR, but the embryos replaced before knowing PGD results. They measured the failure rate of embryos predicted aneuploid by qPCR (negative predictive value) and the success rate of embryos predicted euploid by qPCR (positive predictive value). Embryos that implanted and reached term or miscarried were fingerprinted to be compared wit the originally biopsied embryos. Overall, they found that 96% of euploid embryos failed to sustain implantation and that 41% of euploid embryos had ongoing implantation. Stratifying per type of biopsy, day-3 biopsied embryos had a 29.2% positive predictive value compared to 48.2% (P<0.001) of biopsied blastocysts, while day-3 biopsied embryos had a negative predictive value of 98.1% vs. a 93.5% for biopsied blastocysts.

### Microarray Methods: Clinical Results

Although the trend is away from day-3 biopsy, many clinics are not yet proficient at blastocyst culture and vitrification, which adds extra cost, and a majority of patients prefer to have a fresh cycle. Thus, day-3 biopsy combined with comprehensive chromosome analysis remains the test of choice for these clinics. Others are pushing day 5 biopsy combined with vitrification, specially if using SNP arrays since the technique is not compatible with day 6 replacement. Labs using aCGH or qPCR in the US are increasing the use of day 5 biopsy with replacement on the same cycle, either late day 5 or early day 6. In contrast, in European clinics the tendency is to go back to polar bodies. The clinical results for these different approaches are starting to reported.

CGH is the method with the greatest quantity of clinical data available [6, 64, 65, 74]. Sher *et al.* [64] detected a 74% ongoing pregnancy rate per transfer in women with an average age of 37.5 years after CGH on PBs, however, per cycle started the ongoing pregnancy rate was similar to controls. Schoolcraft *et al.* [6] detected a significant increase in implantation rates, from 46.5% to 72.2% (p<0.001) following blastocyst biopsy, vitrification and embryo selection using CGH. This technique has been mostly discontinued now because is very labor intensive and not automated.

Day 3 biopsy is being performed either with aCGH or SNP arrays. Our most recent data [90] showed that only 78% of PGD cycles had normal embryos for transfer, but if there were normal embryos the ongoing pregnancy rate per transfer was significantly higher than controls (54% vs. 31%, p<0.001). These results are encouraging, but not as impressive as the day-5 (blastocyst) biopsy results.

Day 5 biopsy has recently shown to be less detrimental than day 3 biopsy in a study that compared biopsied but not analyzed embryos. A group of patients received a day 3 biopsied embryos and a non-biopsied embryo, and another group received a blastocyst biopsied embryos and a non-biopsied embryo. The implanting embryos were identified by fingerprinting the biopsied cells. It was observed that while blastocyst biopsy did not affect implantation rates, day 3 biopsy reduced in a 42% the implantation potential of the biopsied embryos. Thus, even a perfect PGD diagnosis could barely improve results since it needs to compensate for this loss of implantation [91].

The use of SNP arrays with blastocyst biopsy and replacement in a thaw cycle produced a significant improvement in pregnancy results [92]. It has been argued that there may be additional advantages associated with transfer in a non-stimulated cycle because of better uterine receptivity [87, 88]. However in an ongoing randomized clinical trial using 24-chromosome analysis by qPCR, higher pregnancy rates were obtained when biopsy was performed on day five and replaced in the same cycle than in controls [91]. Thus it seems that the only difference between day 5 biopsy results and day 3 biopsy results is the detrimental effect of the day-3 biopsy.

Array CGH results also replicate those of SNP and qPCR techniques. (Table **2**) shows some unpublished results from our center showing a significantly higher implantation and ongoing pregnancy rate obtained from day 5 biopsies (either replaced on a thawed cycle or day 6) than day 3 biopsies. 

aCGH is able to differentiate between single chromatid and whole chromosome errors in polar bodies, and a recent study has shown that the majority of meiosis-I aneuploidy errors are caused by chromatids and not whole chromosome errors, with more missing chromosomes or chromatids errors than extra errors [87].

A recent blinded randomized clinical trial comparing blastocyst biopsy and array CGH to a control group, both replaced at day 6, showed significantly better ongoing pregnancy rate (69%) in the PGD group than the control group (42%, p=0.009) [93].

### Microarrays for PGD of Translocations

Recently, aCGH has also been validated for translocations following the same approach used by Gutierrez-Mateo *et al.* [49] of reanalyzing PGD by aCGH diagnosed embryos with FISH. The error rate was 0% when the translocated fragments were 6Mb or larger. aCGH cannot detect at the single cell level translocated fragments below 6Mb, but provided that three of the four translocated fragments are larger than 6Mb, then the cell can be correctly diagnosed as unbalanced or normal/ balanced. Retrospectively looking at over 1000 PGD cycles for translocations showed that none of them had more than one translocated fragment smaller than 6Mb, and thus all translocations can be properly diagnosed by aCGH [88]. The only limitation of aCGH for translocations is that as mentioned above, it cannot differentiate normal from balanced embryos.

## Figures and Tables

**Table 1.** Differences between Whole Chromosome Techniques

	aCGH	SNPs	qPCR	Frequency
**Detection**				
69,XXX w/o other abnormalities	No	Yes	Yes	0.2% *
69,XXX with other abnormalities	Yes	Yes	Yes	7.8% *
Tetraploid w/o other abnormalities	Yes	No	No	n/a
UPD w/o other abnormalities	No	Yes	Unk	> 0.01% **
Meiotic and mitotic duplications w/o recombination	Yes	No	Unk	**3%**
Duplications, Deletions	Yes	Yes	No	5%
Unbalanced Translocations	All	Some	No	Unk
**Other characteristics**				
Parental DNA prior to the test	Unnecessary	Required	Required	
Allow for day 5 biopsy and AM day 6 transfer	Yes	No	Yes	

* ref [82]

** www.ncbi.nlm.nih.gov/omim.

**Table 2.** Results Using Array CGH or SNP Arrays with Either Day 3 or Blastocyst Biopsy

Technique	aCGH	aCGH	SNP Array
Day of Biopsy	Day 3*	Day 5*	Day 5**
Fertility Clinics:	121	23	1
Cycles of PGD:	1089	433	130
Average Maternal Age:	37.0	37.0	37.8
# Embryos Biopsied:	8.3	6.4	5.9
% Euploid Embryos:	31%	49%	47%
*Implantation Rate/Embryo Replaced	39%	61% (52-83%)	65%
*Pregnancies/Cycle	39%	50% (26-73%)	70%
*Pregnancies/Transfer	54%	67% (53-94%)	87%

In brackets is the range per clinic

* (90, 95, and unpublished Reprogenetics results)

** (94)

* p<0.001

## ABBREVIATIONS

aCGH: = Array Comparative Genome Hybridization
ADO: = Allele Drop Out
ART: = Assisted Reproductive Technologies
BAC: = Bacterial Artificial Chromosome
CGH: = Comparative Genome Hybridization
FISH: = Fluorescence In Situ Hybridization
MDA: = Multiple Displacement Amplification
PA: = Preferential Amplification
PB: = Polar Body
OMIM: = Online Mendelina Inheritance in Man
PCR: = Polymerase Chain Reaction
PGD: = Preimplantation Genetic Diagnosis
PGS: = Preimplantation Genetic Screening
qPCR: = Quantitative PCR
SNP: = Single Nucleotide Polymorphism
UPD: = Uniparental Disomy
WGA: = Whole Genome Amplification
